# Fracture rate and time to fracture in dogs with appendicular osteosarcoma receiving finely fractionated compared to coarsely fractionated radiation therapy: A single institution study

**DOI:** 10.1002/vms3.782

**Published:** 2022-03-08

**Authors:** Carissa J. Norquest, Charles A. Maitz, Deborah A. Keys, Melanie Moore, Jeffrey N. Bryan, Tara J. Ehling, Jimmy C. Lattimer, Brian K. Flesner

**Affiliations:** ^1^ Veterinary Health Center University of Missouri Columbia Missouri; ^2^ Kaleidoscope Statistics LLC Athens Georgia; ^3^ Veterinary Health Center at Wentzville University of Missouri Wentzville Missouri; ^4^ School of Veterinary Medicine University of Pennsylvania Philadelphia Pennsylvania

**Keywords:** canine, osteosarcoma, pathologic fracture, radiation therapy, zoledronate

## Abstract

**Background:**

Radiation therapy (RT) is used for local pain alleviation in dogs with appendicular osteosarcoma (OS), especially among dogs that are poor surgical candidates for amputation. However, many historical reports of fractionated protocols lack time to fracture and fracture rates.

**Objectives:**

The primary objectives of this retrospective study were to determine fracture rate and time to fracture of dogs receiving RT (coarse or fine fractionated) for appendicular OS. Secondary objectives were to evaluate tolerability and disease outcome measures.

**Methods:**

Fifty‐one dogs that received RT as part of treatment for appendicular OS were available for evaluation. Forty‐five received coarse fractionation (C‐RT, 8 or 6 Gy per fraction protocols [C‐RT8 or C‐RT6]) while the remaining six received fine fractionation (F‐RT).

**Results:**

The overall pathologic fracture rate was 37%. Pathologic fracture rate was significantly higher for dogs that received F‐RT (5/6, 83%) compared to dogs that received C‐RT (12/40, 30%, *p* = 0.021). In the 17 dogs that fractured, the overall median time to fracture was 57 days. For all dogs, the median progression free interval (PFI) and median overall survival time (OST) were 90 and 140 days, respectively. In a very small cohort of dogs (*n* = 7) treated with zoledronate and RT, fracture rate was 0% and extended survival times were noted.

**Conclusions:**

In conclusion, C‐RT is recommended over F‐RT due to lower risk of pathologic fracture and similar PFI. Prospective evaluation of combined C‐RT and zoledronate, especially for dogs with poor surgical candidacy, is warranted for the treatment of canine appendicular osteosarcoma.

## Abstract 2

Coarse fraction RT is recommended over fine fraction RT due to lower risk of pathologic fracture and similar PFI. Prospective evaluation of combined coarse RT and zoledronate, especially for dogs with poor surgical candidacy, is warranted for the treatment of canine appendicular osteosarcoma.

## 1 INTRODUCTION

Pain relief and local tumour control are necessary to improve quality of life and provide extended survival for dogs with appendicular osteosarcoma (OS). Amputation of the affected limb is currently gold standard as this accomplishes both goals via complete excision. However, a subset of dogs are not good surgical candidates due to pre‐existing co‐morbidities and/or owner reservations regarding amputation. Radiation therapy (RT) has been used for these cases as an alternative primary treatment modality.

The growing availability of RT in veterinary medicine increases patient access to this alternative form of local therapy. Protocols can be broadly characterised by treatment intent or fractionation scheme. Fractionation schemes are grouped by coarse and fine fractionation with coarse fractionation (C‐RT) focused on higher dose per fraction and fine fractionation (F‐RT) prioritising higher total doses. Historically, C‐RT has been associated with palliative intent and F‐RT with curative intent. However, these generalisations have become more complicated as stereotactic body radiotherapy (SBRT), a large dose in substantially fewer fractions using highly conformal dosing, has grown in availability (Coomer et al., 2009).

RT has traditionally been reserved for dogs with appendicular OS in which preservation of the affected limb is prioritised. Pathologic fracture of the affected limb is one of the more severe consequences following RT and is often life‐limiting, especially in dogs for which subsequent amputation is not a viable option. Pathologic fracture rates following RT have been sporadically reported and have ranged from 15% to 63% for different radiation protocols. Reported fracture rates for C‐RT, F‐RT and SBRT are 15–40%, 20% and 36–63%, respectively (Duffy et al., 2018; Fan et al., 2009; Farese et al., 2004; Green et al., 2002; Heidner et al., 1991; Kubicek et al., 2016; McEntee, 1993; Nolan et al., 2020; Oblak et al., 2012; Pagano et al., 2016; Ramirez et al., 1999; Walter et al., 2005). A recent publication describing C‐RT, SBRT and stereotactic radiosurgery (SRS) had an overall pathologic fracture rate of 32%; the fracture rate of each individual protocol was not reported (Nolan et al., 2020). The pathologic fracture rates for F‐RT are derived from two studies; both utilised cisplatin‐based radiosensitisation in all or the majority of patients. These studies do not report time to fracture (Heidner et al., 1991; Walter et al., 2005). Similarly, a subset of C‐RT studies report median times to fracture of 1–8.3 months (Green et al., 2002; Knapp‐Hoch et al., 2009; McEntee, 1993; Pagano et al., 2016; Ramirez et al., 1999). Most of these reports included small sample size and may not reflect a larger population (Green et al., 2002; Knapp‐Hoch et al., 2009; McEntee, 1993; Pagano et al., 2016). Additional confounding factors include uses of different radiation delivery systems and reporting of completed cases versus reporting cases on an intent‐to‐treat basis.

The current retrospective study evaluated dogs that received either C‐RT or F‐RT for appendicular OS on an intent‐to‐treat basis at a single institution. The primary objectives were to determine fracture rate and time to fracture. We hypothesised that F‐RT and bisphosphonate (BP) usage would be associated with a lower rate of pathologic fracture. Secondary objectives included assessing tolerability, progression‐free interval and survival time. To our knowledge, this is the first study to describe dogs with appendicular OS that received C‐RT and F‐RT contemporaneously on an intent‐to‐treat basis at the same institution.

## 2 MATERIALS AND METHODS

### 2.1 Case selection

Electronic medical records were reviewed at the Veterinary Health Center for dogs diagnosed with osteosarcoma that received radiation therapy between 2006 and 2018. Dogs were included if they received radiation therapy following a cytologic, histologic, or clinical diagnosis (radiographic suspicion by board‐certified radiologist) of osteosarcoma of the appendicular skeleton. Dogs were considered to have received radiation therapy on an intent‐to‐treat basis and were not required to have completed their intended protocol for inclusion in the study. Chemotherapy, BPs and additional analgesics were permitted at any point during therapy. All stages of disease were included with no minimum staging requirement. Patients were allowed to have received additional local therapy following RT: additional courses of RT and amputation. Dogs were excluded if the tumour was located on the axial skeleton, that is, was extraskeletal, if they presented with pathologic fracture or if they received radioisotope therapy at any point during treatment. Dogs were also excluded if they did not receive a finely fractionated protocol or one of two coarsely fractionated protocols: 6 × 6 (6 Gy × 6 fractions) or 8 × 4 (8 Gy × 4 fractions): C‐RT6 or C‐RT8.

### 2.2 Radiation therapy

All patients were anaesthetised and immobilised in vacuum cushions (SecureVac, Bionix, Toledo, OH). The vast majority of patients treated with coarse fractionation (C‐RT6 or CRT‐8) were done so using a fixed source‐to‐axis (SAD) distance technique, with dose prescribed to the central axis and monitor units calculated using an accelerator‐specific tissue‐maximum‐ratio (TMR) table. Most patients were treated using parallel‐opposed portals and irradiated volume included the entire lesion radiographically (diagnostic radiographs and MV ports were both evaluated), and at least half the length of the long bone. Most patients receiving finely fractionated radiation therapy were done so using a fixed SAD technique with CT‐based computerised treatment planning using either XiO (Elekta, Stockholm, Sweden) or RayStation (Raysearch Laboratories, Stockholm, Sweden). The gross tumour volume was considered the extent of disease visible on CT scan, including periosteal reaction. A planning target volume (PTV) expansion of 0.5–3.0 cm was included based on the discretion of the radiation oncologist. Treatment plans consisted of two or more beams using a 3D‐CRT technique and the prescribed dose was delivered to 95% of the planning target volume. Positioning was confirmed prior to every treatment with orthogonal MV ports. Tissue‐equivalent bolus was used as necessary at the discretion of the radiation oncologist; when used, bolus was included in monitor unit and dose calculations. X‐ray irradiation was performed using either a Siemens Mevatron, Siemens Oncor (Siemens AG, Munich, Germany), or Varian 21EX (Varian, Palo Alto, CA) linear accelerator. All activities were performed in accordance with daily, monthly and annual quality assurances, performed by AAPM‐certified medical physicists.

### 2.3 Medical record review

Medical record review for follow‐up was performed by one clinician (CJN) in a standardised fashion; this was overseen by one senior clinician (BKF). Patient information abstracted from the medical record included signalment (age, sex, breed, weight), presenting complaint, duration of clinical signs, tumour location, ALP status at diagnosis, TNM stage, radiation side effects, previous and concurrent therapies and radiation therapy plan. Radiation side effects based on the Veterinary Radiation Therapy Oncology Group (VRTOG) toxicity scoring scheme (Ladue & Klein, 2001) were either obtained directly from the medical record or were retrospectively graded based off the details in the medical record; all dogs had a 2‐week follow‐up visit (either at the academic institution or with their primary veterinarian) unless not completing their RT protocol. A standardised follow‐up protocol after initial 2‐week recheck was not present. Side effects were deemed ‘late’ if occurring 6 months after completion of RT. Patients were considered to have received chemotherapy if they received chemotherapy at any point during overall treatment for their disease. Determination for type of RT protocol received was based on the number of fractions initially prescribed. C‐RT was defined as protocols consisting of 6 fractions or less whereas F‐RT protocols consisted of at least 18 prescribed fractions. Type of RT (F‐RT versus C‐RT) was chosen based on owner and clinician preference.

Pathologic fracture occurrence was concluded if stated explicitly in the medical record (referring veterinarian confirmation by radiographs, histopathologic confirmation after amputation) or in attached radiology reports signed by board‐certified veterinary radiologists. Pathologic fractures were considered absent in cases with adequate follow‐up (i.e. dogs continued to return to our institution or to their primary veterinarian for routine monitoring and/or follow‐up after finishing their course of RT). Patients were censored from pathologic fracture evaluation if the medical record was incomplete.

Response to therapy (from the start of RT) was determined subjectively through review of medical records evaluating for improved lameness, comfort and attitude. Time to progression was defined as time from first radiation therapy treatment to progressive disease defined as local tumour progression (pain, lameness, swelling etc.) or development of metastatic lesions. Patients were censored from disease progression analysis if they were lost to follow‐up or did not have documented progression at completion of data accrual.

Survival time was measured in days from time of first radiation therapy treatment to date of death in the medical record. Causes of death were categorised as due to local disease, metastatic disease or non‐tumour related. Cause of death was recorded as tumour‐related local disease when it was unknown. Additional follow‐up information was obtained by contacting the referring veterinarian listed in the patient file. Patients were censored from survival analysis if they were lost to follow‐up or were still alive at completion of the study.

### 2.4 Statistical analysis

All analyses were performed using SAS V 9.4 (Cary, NC). A significance threshold of 0.05 was used. Exact binomial confidence intervals were calculated for percentages. Fisher's exact tests were used to compare presence of fractures or side effects (yes/no) between radiation groups and also presence of fractures between bisphosphate groups. Dose per fraction was compared between dogs with and without fractures by a Mann–Whitney *t*‐test. The Kaplan–Meier method was used to construct survival curves and median survival estimates with 95% confidence intervals for PFI and OST. Log‐rank tests were used to compare PFI and OST between strata and were adjusted for multiple comparisons with a Hochberg adjustment. Cox proportional hazards regression was used to test total (delivered) dose, number of fractions and dose per fraction for association with PFI and OST.

## 3 RESULTS

Electronic medical record review returned 120 dogs with 51 dogs meeting inclusion criteria. Thirty‐one dogs were excluded for extraskeletal or axial skeleton tumour locations. Twenty‐six dogs were excluded for receiving coarsely fractionated protocols other than 6 or 8 Gy fractions. Twelve dogs were excluded for receiving radioisotope therapy. Diagnosis of OS was made via cytology or histopathology in 28 dogs and was clinically presumed in the remaining 23 dogs based on radiologic confirmation of an aggressive bone lesion by a board‐certified radiologist (JCL). No pathologic fractures were noted in these 51 dogs prior to initiating treatment. This was based on imaging for RT planning purposes. Forty‐five dogs had limb radiographs (*n* = 3 F‐RT, *n* = 42 C‐RT) while six dogs had CT guided plans (*n* = 3 F‐RT, *n* = 3 C‐RT). At presentation, the median age of all dogs was 8 years (range: 5–14 years), and the median body weight was 48.2 kg (range: 27.2–82.1 kg). There were 2 intact male dogs, 26 male neutered dogs, 1 intact female dog and 22 spayed female dogs. The male to female ratio was 1.22:1. There were a total of 20 breeds represented with the most common breeds being Golden retriever (*n* = 9) and Great Dane (*n* = 7). Additional common breeds included Great Pyrenees (*n* = 4), German Shepherd (*n* = 3), Labrador retriever (*n* = 3), Mastiff (*n* = 3), Rottweiler (*n* = 3) and mixed breed dogs (*n* = 3); two patients or fewer comprised the remaining breeds. The most common presenting complaints were lameness (48/51, 94%), swelling (26/51, 51%) and pain (16/51, 31%). The median duration of clinical signs prior to presentation was 30 days (range: 5–210 days). The most common tumour location was the radius (28/51, 53%) followed by the humerus (13/51, 25%), femur (5/51, 10%), tibia (4/51, 8%) and scapula (1/51, 2%).

Staging was not standardised. Distant metastasis (M stage) was defined for 49/51 dogs based on physical exam and thoracic radiographs or thoracic computed tomography scan, and rarely nuclear medicine bone scan; 10/49 dogs had distant metastasis (bone or lung) present at treatment initiation (F‐RT = 1 [lung], C‐RT = 9; simultaneous primary and metastatic bone lesions [*n* = 3], metastatic to new bone in dog with previously amputated limb [*n* = 3], lung [*n* = 3]). All metastatic bone lesions were irradiated with the same protocol chosen for the primary tumour. Metastatic lung was not treated with RT. Two dogs did not have thoracic imaging available for review.

Tables 1A and B describe treatment groups and pre‐treatment characteristics of the treatment groups. The completion rate for coarse (C‐RT) and fine (F‐RT) fractionation protocols were 87% and 67%, respectively. The total dose for C‐RT ranged from 8 to 36 Gy over 1 to 40 days, depending on completion of the protocol. Most (*n* = 33) dogs in the C‐RT arm received 8 Gy fractions administered weekly for 4 weeks for a total of 32 Gy (C‐RT8); the remaining dogs (*n* = 12) were prescribed 6 Gy fractions administered weekly for 6 weeks for a total of 36 Gy (C‐RT6). Dogs receiving F‐RT were prescribed 18 or 19 fractions at a range of 3–3.25 Gy per fraction for an expected total dose of 57–58.5 Gy. As two dogs fractured in the early portion of their F‐RT, they only received 3.2 and 17.5 Gy, respectively. Median duration of RT was 28 days for the 4 dogs that completed the full course of F‐RT.

The overall pathologic fracture rate was 37% (17/46). Five dogs were censored from this analysis as their medical records could not verify or nullify a fracture. These five dogs’ median time of follow‐up was 30 days (range: 22–88 days). Weight was not associated with fracture; for example, in the five heaviest dogs (> 70 kg), only 1/5 dogs developed a pathologic fracture. Fracture rates were similar across tumour locations (radius 32%, humerus 31%, tibia 25%) except for the femur (3/5, 60%). The fracture rate in the F‐RT group was 83% (5/6) which was significantly higher compared to 30% (12/40) in the C‐RT group (*p* = 0.021). For individual arms of the C‐RT group, the fracture rate for C‐RT8 was 30% (9/30) which was the same as 30% (3/10) for C‐RT6. The fracture rate in the no BPs group was 47% (7/15) compared to 42% (10/24) in the pamidronate group and 0% (0/7) in the zoledronate group (*p* = 0.094 comparing all three groups; *p* = 0.066 comparing pamidronate vs. zoledronate). This data is summarised in Table 2. The mean dose per fraction in dogs that fractured was 6.22 Gy which was not significantly different than the mean in dogs that did not fracture, 7.35 Gy (*p* = 0.097). In the 17 dogs that fractured, the overall median time to fracture (TTF) was 57 days. The median TTF for C‐RT and F‐RT were 46 and 111 days, respectively, and were not statistically different (*p* = 0.80).

**TABLE 2:** Effect of bisphosphonates on fracture rate

Bisphosphonate	‘*n*’ fractured / ‘*n*’ in group	Fracture rate
None	7/15	47%
Pamidronate	10/24	42%
Zoledronate	0/7	0%

The overall acute side effect percentage was 38% (17/45) and the late side effect proportion was 36% (9/25). The acute side effect percentage in F‐RT was 50% (2/4) compared to 37% (15/41) in C‐RT. Given the appendicular tumour location, all recorded acute side effects were limited to skin with 55%, 18% and 27%, respectively graded as VRTOG 1, 2 and 3. The majority of VRTOG side effects were dry desquamation and alopecia. Of the dogs reported to have VRTOG scores >2 or 3, 80% were attributed to self‐trauma to the area. The remaining 20% were suspected to be experiencing local tumour progression based on clinical signs. The late side effect percentage in the F‐RT group was 0% (0/4); late side effect percentage in the C‐RT group was 43% (9/21). Late side effects were only documented for the skin in which over 70% of lesions were graded as VRTOG 1 (alopecia and leukotrichia) with the remaining graded as VRTOG 3. There were no statistical differences in toxicity profile for C‐RT8 versus C‐RT6.

**FIGURE 1:**
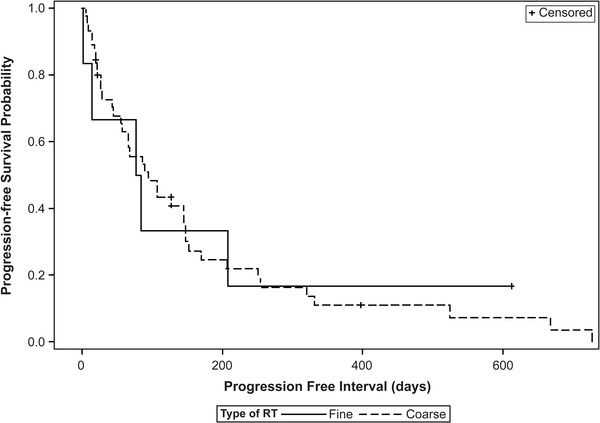
Progression free interval does not differ between radiation types. Dogs receiving fine (solid line, median PFI = 80.5 days) versus coarse (hashed line, median PFI = 96 days) fraction radiation therapy did not have statistically significantly different progression free intervals (*p* = 0.943). Censored cases are noted by tick marks

Overall median progression free interval (PFI) was 90 days. Seven dogs were censored from PFI analysis because of lost to follow‐up (*n* = 3), death from other cause (*n* = 2: progression of unrelated lymphoma; immune‐mediated thrombocytopenia [ITP] with seizures), incomplete medical records (*n* = 1) or still alive at time of data collection (*n* = 1). Factors evaluated for PFI are summarised in Table 3. PFI differed based on tumour location with radius having the longest PFI. PFI did not differ significantly between F‐RT (median 80.5 days) and C‐RT (median 96 days, *p* = 0.943). Kaplan–Meier PFI curves are shown in Figure 1. However, when split into C‐RT8 vs. C‐RT6, a statistically significant increased PFI was noted in dogs receiving an 8 Gy/fraction protocol; median PFI for C‐RT8 was 146 days versus 50.5 days for C‐RT6 (*p* = 0.020). The median (95% CI) PFI of dogs with normal ALP at diagnosis was 137 (27–208) days compared to 67 (22–146) days (*p* = 0.055) in dogs with elevated ALP at diagnosis. PFI was significantly increased if lameness improved within 30 days of RT (*p* = 0.007), completing the prescribed RT protocol (*p* < 0.001), and receiving any chemotherapy (*p* = 0.003). Unfortunately, due to the retrospective nature of this study, chemotherapy protocol was not standardised. Chemotherapeutics administered included carboplatin, doxorubicin and toceranib, with most dogs receiving either a single agent carboplatin or carboplatin/doxorubicin protocol. PFI was not increased by using a BP (*p* = 0.185). Of note, timing of BP administration in respect to RT (neo‐adjuvant, concurrent or adjuvant) was not standardised.

**TABLE 3:** Median progression free interval (PFI) by factors

Factor	Factor (*n*)	Median PFI (95% CI)	*p* Value^a^
Pain (0 = none, 1 = pain or lameness)	0 (3)	206 (66–332)	0.620
	1 (48)	86 (56–146)	
ALP (0 = WNL, 1 = increased)	0 (22)	137 (27–208)	0.055
	1 (20)	67 (22–146)	
Tumour location (1 = radius, 2 = humerus, 3 = tibia, 4 = femur, 5 = other such as scapula/ulna)	1 (28)	146 (67–206)	0.018
	2 (13)	86 (22–148)	
	3 (4)	44 (2–332)	
	4 (5)	44 (9–90)	
	5 (1)	NC	
Treated tumour (1 = primary tumour, 2 = metastatic lesion)	1 (43)	86 (56–153)	0.164
	2 (8)	98.5 (2–146)	
M stage	0 (39)	84 (45–153)	0.238
	1 (10)	98.5 (2–146)	
Radiation (0 = F‐RT, 1 = C‐RT)	0 (6)	80.5 (2–NC)	0.943
	1 (45)	96 (56–148)	
Radiation (0 = F‐RT, 1 = C‐RT8, 2 = C‐RT6)	0 (6)	80.5 (2–NC)	0.785 (0 vs. 1)
	1 (33)	146 (68–153)	0.443 (0 vs. 2)
	2 (12)	50.5 (15–86)	0.020 (1 vs. 2)
RT plan (0 = manual, 1 = computer)	0 (45)	90 (45–148)	0.860
	1 (5)	86 (14–255)	
Completed RT (0 = no, 1 = yes)	0 (8)	11.5 (2–19)	<0.001
	1 (43)	128 (77–153)	
Improvement w/n 30 days of RT (0 = no, 1 = yes)	0 (23)	29 (19–68)	0.007
	1 (28)	146 (86–251)	
Chemotherapy (0 = none, 1 = any)	0 (15)	29 (15–68)	0.003
	1 (34)	137 (77–170)	
Bisphosphonates (0 = none, 1 = pamidronate, 2 = zoledronate)	0 (18)	44 (22–86)	0.381 (1 vs. 2 vs. 3) 0.185 (0 vs. 1+2)
	1 (26)	128 (67–148)	
	2 (7)	170 (14–251)	
NSAID (0 = none, 1 = yes)	0 (9)	86 (22–251)	0.580
	1 (42)	90 (58–148)	

^a^ Log‐rank test.

NC = noncalculable; F‐RT = finely fractionated; C‐RT = coarsely fractionated; C‐RT8 = 8 Gy per fraction; C‐RT6 = 6 Gy per fraction.

**FIGURE 2:**
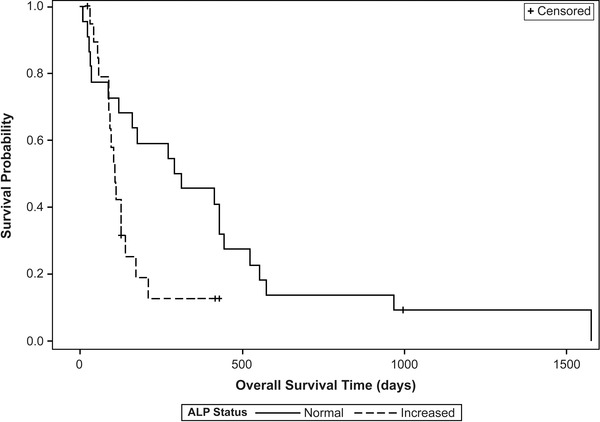
Overall survival time is increased by having normal ALP status. Dogs with normal serum ALP (solid line, MST = 301 days) had a significantly longer median survival time than dogs with increased serum ALP (hashed line, MST = 108 days), *p* = 0.033. Censored cases are noted by tick marks

**FIGURE 3:**
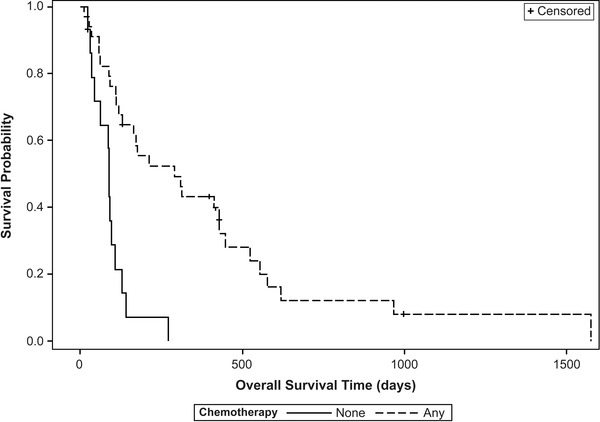
Overall survival time is increased by receiving chemotherapy. Dogs receiving any chemotherapy agent (hashed line, MST = 291 days), had a significantly longer median survival time than dogs who did not receive chemotherapy (solid line, MST = 88 days), *p* < 0.001. Censored cases are noted by tick marks

Median overall survival time (MST) for all cases was 140 days. Seventeen dogs were censored from analysis (F‐RT 3/6, C‐RT 14/45). In the F‐RT group, one dog died of unrelated causes (seizures and ITP) and two dogs were lost to follow‐up. In the C‐RT group, four dogs died of non‐tumour causes (progression of lymphoma, progression of abscess, septic neutropenia, suspect renal failure), eight dogs were lost to follow‐up, one died from unknown causes and one was still alive. Of tumour‐related deaths, 19 (F‐RT = 1, C‐RT = 18) were due to local progression/disease based on progressive clinical signs and 15 (F‐RT = 2, C‐RT = 13) were due to metastatic disease (distant lesions confirmed via physical exam with cytology/histopathology or imaging with radiographs/CT). Factors affecting OST are summarised in Table 4. While dogs receiving F‐RT (MST 454 days) had a longer survival compared to dogs receiving C‐RT (127 days), the *p*‐value (*p* = 0.055) is marginally above the 0.05 threshold; the data neither establish a conclusive difference nor a conclusive similarity of survival between C‐RT and F‐RT. When evaluating the two C‐RT groups, dogs receiving C‐RT8 had a significantly longer MST than dogs receiving C‐RT6 (171 days compared to 74.5 days, *p* = 0.002). There was no difference in survival of F‐RT dogs compared to C‐RT8 dogs (*p* = 0.113). Increased OST was associated with a normal ALP at diagnosis (*p* = 0.033, Figure 2), lameness improvement within 30 days of RT (0.019), completing the prescribed RT protocol (0.027), and receiving any chemotherapy (*p* < 0.001, Figure 3). Increased OST was not associated with receiving any BP (*p* = 0.234). Notably, survival was significantly different between BP/radiation groups (*p* = 0.026). These survival times are summarised in Table 5. In seven dogs treated with C‐RT protocols and zoledronate, median survival time was 445 days with no fractures reported. However, in a post hoc comparison, zoledronate was not associated with improved progression free interval or overall survival time, compared to the other BP groups (pamidronate and no bisphosphonate): *p* = 0.436 and *p* = 0.256, respectively.

**TABLE 4:** Median overall survival time (MST) by factors

Factor	Factor (*n*)	MST (95% CI)	*p* Value^a^
Pain (0 = none, 1 = pain or lameness)	0 (3)	412 (111‐NC)	0.474
	1 (48)	127 (89–211)	
ALP (0 = WNL, 1 = increased)	0 (22)	301.5 (87–445)	0.033
	1 (20)	108 (88–140)	
Tumour location (1 = radius, 2 = humerus, 3 = tibia, 4 = femur, 5 = other such as scapula/ulna)	1 (28)	177 (96–429)	0.546
	2 (13)	116 (43–308)	
	3 (4)	261.5 (33‐NC)	
	4 (5)	58 (9–NC)	
	5 (1)	NC	
Treated tumour (1 = primary tumour, 2 = metastatic lesion)	1 (43)	140 (89–291)	0.570
	2 (8)	149 (43–312)	
M Stage	0 (39)	140 (89–412)	0.402
	1 (10)	149 (43–271)	
Radiation (0 = F‐RT, 1 = C‐RT)	0 (6)	454 (118–NC)	0.055
	1 (45)	127 (88–176)	
Radiation (0 = F‐RT, 1 = C‐RT8, 2 = C‐RT6)	0 (6)	454 (118–NC)	0.113 (0 vs. 1)
	1 (33)	171 (105–412)	0.006 (0 vs. 2)
	2 (12)	74.5 (30–89)	0.002 (1 vs. 2)
RT plan (0 = manual, 1 = computer)	0 (45)	127 (89–177)	0.333
	1 (5)	291 (87–NC)	
Completed RT (0 = no, 1 = yes)	0 (8)	31 (9–291)	0.027
	1 (43)	162 (108–308)	
Improvement w/n 30 days of RT (0 = no, 1 = yes)	0 (23)	92 (35–176)	0.019
	1 (28)	211 (118–429)	
Chemotherapy (0 = none, 1 = any)	0 (15)	88 (35–105)	<0.001
	1 (34)	291 (118–429)	
Bisphosphonates (0 = none, 1 = pamidronate, 2 = zoledronate)	0 (18)	62 (33–271)	0.344 (1 vs. 2 vs. 3) 0.234 (0 vs. 1+2)
	1 (26)	162 (108–312)	
	2 (7)	445 (89–575)	
NSAID (0 = none, 1 = yes)	0 (9)	87 (27–445)	0.152
	1 (42)	162 (96–308)	

^a^ Log‐rank test.

NC = noncalculable; F‐RT = finely fractionated; C‐RT = coarsely fractionated; C‐RT8 = 8 Gy per fraction; C‐RT6 = 6 Gy per fraction.

**TABLE 5:** Median overall survival time by bisphosphonate and radiation type

Bisphosphonate	Fractionation	(*n*)	Median (95% CI)
None	F‐RT	4	454 (271–617)
None	C‐RT	14	58 (30–85)
Pamidronate	F‐RT	2	NC
Pamidronate	C‐RT	24	162 (105–312)
Zoledronate	C‐RT	7	445 (89–575)

*Note*: Log‐rank, *p* = 0.026.

NC = not calculable; F‐RT = finely fractionated; C‐RT = coarsely fractionated.

**TABLE 1A:** Treatment groups

Variable	Type of RT	*n*	Median	Lower quartile	Upper quartile	Min.	Max.
Dose/fraction (Gy)	F‐RT	6	3.125	3.0	3.20	3.0	3.25
	C‐CT	45	8.0	6.0	8.0	6.0	8.0
Number of fractions	F‐RT	6	18	9	18	1	19
	C‐CT	45	4	4	4	1	6
Total dose (Gy)	F‐RT	6	55.5	27.5	57.5	3.2	58.5
	C‐CT	45	32	32	32	8	36

**TABLE 1B:** Pre‐treatment characteristics

Factors		F‐RT		C‐RT	
		*N*	%	*N*	%
Pain or lameness	Yes	6	100	42	93.3
	No	0	0	3	6.7
Tumour location	Radius	4	66.7	24	53.3
	Humerus	0	0	13	28.9
	Tibia	1	16.7	3	6.7
	Femur	1	16.7	4	8.9
	Other (scapula)	0	0	1	2.2
Treated tumour	Primary	5	83.3	38	84.4
	Metastatic	1	16.7	7	15.6
Distant metastasis (M1 Status)	Yes	1	16.7	8	18.6
	No	5	83.3	35	81.4
Type of radiation	Fine	6	100	0	0
	C‐RT8	0	0	33	73.3
	C‐RT6	0	0	12	26.7
RT plan	Computer	3	50	2	4.5
	Hand	3	50	42	95.5
Completed RT	Yes	4	66.7	39	86.7
	No	2	33.3	6	13.3
Improvement w/n 30 days of RT	Yes	3	50	25	55.6
	No	3	50	20	44.4
Chemotherapy	Yes	5	83.3	29	67.4
	No	1	16.7	14	32.6
Bisphosphonates	None	4	66.7	14	31.1
	Pamidronate	2	33.3	24	53.3
	Zoledronate	0	0	7	15.6
NSAID	Yes	6	100	36	80
	No	0	0	9	20
ALP status	Increased	1	20	19	51.4
	Normal	4	80	18	48.6

## 4 DISCUSSION

The middle‐aged to older large‐breed patient population in this study is similar to those in previous studies. The most commonly recorded breeds (Golden retriever, Great Dane, Labrador retriever, Mastiff, Rottweiler and mixed breed) are consistent with previously reported predisposed breeds. Sex predisposition has been documented in some studies while others did not validate this finding. (Tuohy et al., 2019) The median duration of clinical signs prior to presentation (30 days) was similar to the only previous report of 15 days. (Pagano et al., 2016) The most common tumour location was the radius (55%), consistent with a large, previous study. (Tuohy et al., 2019) The percentage of M1 stage patients (20%) in this study likely reflects selection bias, as patients with existing gross metastatic disease would be more likely to be directed towards palliative than definitive care. Only one M1 stage patient was prescribed F‐RT; this dog had a suspected skip metastasis on bone scan from the distal right tibial OS to the mid‐diaphyseal region of the right tibia.

The overall pathologic fracture rate in this study (37%) was similar to previous reports (Boston et al., 2007; Coomer et al., 2009; Duffy et al., 2018; Fan et al., 2009; Farese et al., 2004; Green et al., 2002; Heidner et al., 1991; Kubicek et al., 2016; McEntee, 1993; McEntee, 1997; Nolan et al., 2020; Oblak et al., 2012; Pagano et al., 2016; Ramirez et al., 1999; Walter et al., 2005). Patients who received F‐RT had higher rate of fracture (83%); this was significantly higher than the C‐RT group. This did not support our hypothesis that F‐RT would be associated with a lower fracture rate. Bone is a late responding tissue and is more sensitive to higher doses per fraction as delivered in C‐RT and SBRT protocols (Coomer et al., 2009). Fracture was not associated with a higher dose per fraction. VRTOG late RT bone effects include pain on palpation, radiographic changes and necrosis, with the latter two potentially associated with pathologic fracture following RT (Ladue & Klein, 2001). F‐RT should result in less bone necrosis and a decreased incidence of pathologic fracture. However, pathologic fractures are also commonly the result of local tumour progression causing decreased integrity and increased necrosis of the bone. It is possible that the dose per fraction of F‐RT is inadequate for local control of a low α:β tumour such as OS (Gillette et al., 1995; Nolan & Gieger, 2019). Two F‐RT dogs fractured within 4 and 14 days after starting their protocol.

Our second hypothesis that bisphosphonate therapy would be associated with a decreased incidence of pathologic fracture was also rejected. The fracture rate for dogs that did not receive bisphosphonates was 47% compared to 42% and 0% for dogs that received pamidronate and zoledronate, respectively. Unfortunately, as stated above, timing of BP administration in relation to RT was not standardised. Bisphosphonates have been shown to significantly decrease skeletal morbidity in human cancer patients with bone metastases (Clezardin, 2013; Ross, 2003; Vassiliou & Kardamakis, 2009). The skeletal benefit of bisphosphonates is related to their localisation in remodelling bone and reduction of pathologic bone resorption by osteoclasts. Zoledronate is a third‐generation aminobisphosphonate, with several appealing qualities compared to its second‐generation predecessor, pamidronate. It has the benefit of a shorter administration time (15 minutes versus 2 h) and 100‐fold increase in anti‐resorptive potency (Fan, 2007). Zoledronate was first administered at our institution in 2016, which could introduce a temporal bias. Also, only a small number of dogs (*n* = 7) in this study received zoledronate.

Overall median time to fracture (TTF) for this study was 1.9 months. As discussed previously, median TTF has been inconsistently reported from 1 to 8.3 months for C‐RT to 5.8 to 5.9 months for SBRT/SRS (Farese et al., 2004; Kubicek et al., 2016; Pagano et al., 2016). An extended median TTF of 20.9 months was recently reported for dogs undergoing C‐RT, SBRT and SRS; however, fracture rate and TTF was not distinguished between the 3 groups (Nolan et al., 2020). To our knowledge, this is the first study to report median TTF for dogs undergoing F‐RT (3.7 months) for appendicular OS. We censored five dogs (<10% of our study population) from fracture analysis due to loss of follow‐up. As these dogs’ median time to follow‐up was 30 days, it is possible they could have developed fractures. A prospective study with standardised follow‐up would more accurately define fracture rate and time to fracture.

As dogs (*n* = 6, all C‐RT) with potential bone metastasis (either multiple bone lesions at diagnosis or bone metastasis after amputation for primary tumour control) were included in our study, weight bearing and risk of fracture were not equal among the population. Three treatment‐naïve dogs and three dogs with amputation for a primary tumour had bone ‘metastasis’. The three treatment naïve dogs included one dog with bilateral humeral lesions, one dog with bilateral radial lesions and one dog with a distal radius lesion and metastasis to the contralateral scapula; all three received the same C‐RT protocol to both lesions. The remaining three C‐RT dogs had a novel bone metastasis noted after previous amputation for the original primary tumour. In this subset of dogs, 2/6 developed pathologic fracture (1/3 treatment naïve, 1/3 novel bone lesion after amputation). Additionally, the remaining 2/3 dogs with prior amputation had progressive disease and little benefit from C‐RT.

Fracture rate and TTF have serious implications for long‐term outcome for dogs with appendicular OS undergoing RT. Many dogs may be euthanised as result of pathologic fractures. Published rates of euthanasia following pathologic fracture range from 38% to 50% with the remaining patients undergoing amputation (Kubicek et al., 2016; Ramirez et al., 1999). While there are reported positive outcomes of fracture stabilisation following RT, it is typically associated with high complication rate and poor long‐term limb function (Boston et al., 2017; Farese et al., 2004; Thrall et al., 1990).

Incidence of side effects, both acute and late, were not statistically significant between C‐RT and F‐RT. Applying VRTOG scores to RT for primary bone tumours is challenging as a degree of necrotic bone is likely present in both untreated and treated OS (Powers et al., 1991). As the VRTOG scoring scheme was designed to objectively evaluate radiation effects on normal tissues, effects on neoplastic bone should not be graded. Pathologic fracture was not included as a late effect in this study as it is not explicitly stated as a late effect of RT in the VRTOG Late Radiation Morbidity Scoring Scheme (Ladue & Klein, 2001). Furthermore, the retrospective nature of this study precludes standardised follow‐up so it is likely that the true incidence of side effects is misreported. It is possible that lower VRTOG scores were underreported as these rarely raise quality of life concerns. On the other hand, it is possible the current patient population did not live long enough to develop additional late side effects. Improved survival times following RT may increase the reported frequency of late side effects. The increasing use of RT for appendicular OS generates the need for a consensus on scoring late RT effects on bone.

The overall median PFI (3 months) in this study is consistent with previous reports with PFI ranging from 1.8 to 6.7 months. Factors evaluated for PFI are summarised in Table 3. A potential limitation of our retrospective study was that other factors (i.e. infection, orthopaedic disease etc.) that could also cause lameness, swelling or pain were not always completely ruled out, possibly affecting our PFI. Alternatively, dogs could've had progression without overt changes in clinical signs, artificially prolonging our reported PFI. Radial location was associated with an increased PFI (4.9 months). Distal radius location has been documented previously as a positive prognostic indicator for survival (Knapp‐Hoch et al., 2009). In another retrospective paper, however, proximal humerus location was associated with a significantly longer duration of response compared to all other locations (Ramirez et al., 1999). PFI was not significantly influenced by radiation protocol. The F‐RT PFI in this study is the lowest reported with previous F‐RT PFI reported as 5.9 and 6.7 months. Of note, both of the previous studies used intra‐arterial cisplatin (*n* = 12/12) or biodegradable cisplatin implant (*n* = 9/11) as additional local therapy (Heidner et al., 1991; Walter et al., 2005). Other studies utilised F‐RT prior to limb‐spare surgeries and are not considered comparable to RT alone (LaRue et al., 1989; Thrall et al., 1990). Therefore, this likely represents the first study to report PFI for dogs undergoing F‐RT alone for appendicular OS.

Median overall survival time (MST) of 4.7 months is on the lower end of reports of canine appendicular OS treated with any type of RT (3–12.1 months). SRS and SBRT may result in longer MST (9.7–12.1 months). Reports of C‐RT protocols are shorter (3–10.4 months) with the longest MST documented by Green et al. (2002). Retrospectively generated OST may be biased by willingness of owners to pursue additional therapy following complications. In one study, amputation following surgical stabilisation and SBRT/SRS was pursued in half of the patients (*n* = 9) at a median of 5‐month following RT. This cohort was considered at a high risk of fracture at initiation of the study and went on to experience a complication rate of 94%, with 88% of those considered major complications (Boston et al., 2017). Therefore, it may be that more expensive, curative intent RT protocols (F‐RT, SRS and SBRT) select for owners that can afford and are more willing to pursue additional therapy. The same bias is likely reflected in the current study, as receiving F‐RT was associated with longer survival in the face of an increased pathologic fracture rate and similar PFI compared to C‐RT. The two dogs that fractured during F‐RT were subsequently amputated for an overall amputation rate following RT of 40% for F‐RT. Comparatively, only 7% of dogs were amputated following loss of local tumour control with C‐RT. This may also reflect clinician selection bias unlikely to recommend amputation for patients with more advanced disease. A recent study reported a median survival of 205 days following secondary amputation following limb‐spare complications so it is not unreasonable to apply similar reasoning to secondary amputation following RT (Wustefeld‐Janssens et al., 2020). Additional factors evaluated against patient survival are summarised in Table 4.

Dogs receiving C‐RT and zoledronate (14.8 months) had a longer MST than C‐RT dogs that received pamidronate (5.4 months) or dogs that did not receive any bisphosphonate (1.9 months). A previous multi‐cohort retrospective study identified that the addition of pamidronate to RT (with or without chemotherapy) negatively affected survival (Oblak et al., 2012). Pamidronate was historically used at our institution due to the inaccessibility of newer bisphosphonate agents like zoledronate. Due to zoledronate's current availability and cost‐effectiveness, this bisphosphonate is now used as non‐surgical standard of care in our veterinary patients with bone malignancies.

Positive prognostic factors shared between PFI and OST were normal ALP at diagnosis, improvement within 30 days of RT, completion of RT and use of chemotherapy at any point. Increased ALP at diagnosis for dogs with appendicular OS has been a well‐documented negative prognostic factor for survival (Boerman et al., 2012), but has not been prognostic in some studies evaluating RT (Duffy et al., 2018; Mueller et al., 2005; Walter et al., 2005). Previous studies have stressed the importance of case selection for RT with dogs that respond to RT having better long‐term outcomes (Bateman et al., 1994; Farese et al., 2004; Kubicek et al., 2016; McEntee, 1997; Oblak et al., 2012). Understanding ideal RT candidates is still a work in progress within veterinary medicine. Better indicators of RT benefit are needed. We relied on subjective interpretations by treating veterinarians and client owners due to our retrospective study design. To improve objective outcome measures of pain control, our group performed a pilot study using multi‐modal pain assessment (Flesner et al., 2021). Future prospective studies evaluating the success of interventional therapies in reference to pain relief should use objective indicators.

The use of chemotherapy significantly increased both PFI and OST in our study; 5/6 dogs in the F‐RT group and 29/45 dogs in the C‐RT received some form of chemotherapy (majority carboplatin‐based; others included doxorubicin and toceranib). However, as this was a retrospective study, type, dosage and timing of chemotherapy was not standardised and was heavily dependent on clinician preference. This verifies the findings of several previous studies that also found the implementation of chemotherapy resulted in improved outcomes (Farese et al., 2004; Nolan et al., 2020; Oblak et al., 2012; Ramirez et al., 1999). Other reports have not documented an outcome advantage associated with chemotherapy (Duffy et al., 2018; Green et al., 2002; Mueller et al., 2005; Walter et al., 2005). Carboplatin is a known radiosensitiser (Rejec et al., 2015). However, the likelihood of carboplatin improving RT efficacy due to radiosensitisation in our study is unlikely as most dogs received adjuvant chemotherapy after RT was administered for local control.

Our study is inherently limited by its retrospective design. The patient numbers in the F‐RT group are low making it challenging to have definitive comparisons between the protocol types. Adjuvant therapies (chemotherapy, bisphosphonate therapy and analgesics) were not randomised or standardised among our population. A multivariable analysis to adjust for possible confounding factors was not performed due to the low sample size of the F‐RT group (*n* = 6). As previously mentioned, selection bias may greatly affect patient selection as cases with clinically negative factors, such as increased lameness or higher M stage, would be more likely to receive palliative care. In general, RT may be considered palliative contributing to a lower survival time for the overall population, and C‐RT may comprise a further palliative subset. Additionally, owner finances and perception of quality of life play substantial roles in case progression. At our institution, C‐RT is roughly half the cost compared to F‐RT which may select for more financially limited owners that are less likely to pursue additional adjuvant therapies. Additionally, owners that elect for a more palliatively designed protocol, such as C‐RT, may be less likely to tolerate any clinical signs that reflect decreased quality of life resulting in earlier euthanasia times. Follow‐up was not standardised and was based on review of medical records and referring clinic contact inserting a component of recall bias.

In summary, C‐RT is recommended over F‐RT due to lower pathologic fracture rate and a similar PFI documented in the study population. Chemotherapy improved OST in this population and should be considered for dogs undergoing C‐RT for appendicular OS. Further enhancement of current RT protocols for canine osteosarcoma patients that are not suitable candidates for standard of care treatment is necessary. Additional prospective clinical studies investigating the role of zoledronate combined with C‐RT protocols for dogs with primary appendicular OS are warranted.

## ETHICS STATEMENT

The authors confirm that the ethical policies of the journal, as noted on the journal's author guidelines page, have been adhered to. No ethical approval was required as this is a retrospective study where standard of care was offered to client owners.

## FUNDING INFORMATION

The authors received no funding for this article.

## AUTHOR CONTRIBUTIONS

Deborah A. Keys: formal analysis; writing – original draft; writing – review & editing. Melanie Moore: data curation; investigation; writing – review & editing.

## CONFLICT OF INTEREST

The authors have no conflicts of interest.

### PEER REVIEW

The peer review history for this article is available at https://publons.com/publon/10.1002/vms3.782

## Data Availability

Data is available by reasonable request(s) to the corresponding author.
